# Translational simulation: from description to action

**DOI:** 10.1186/s41077-021-00160-6

**Published:** 2021-03-04

**Authors:** Christopher Peter Nickson, Andrew Petrosoniak, Stephanie Barwick, Victoria Brazil

**Affiliations:** 1 Intensive Care Unit and Centre for Health Innovation, Alfred Health, Melbourne, Australia; 2 School of Public Health and Preventative Medicine, Monash University, Melbourne, Australia; 3 St. Michael’s Hospital, Toronto, Canada; 4 Department of Medicine, University of Toronto, Toronto, Canada; 5 Mater Education, South Brisbane, Queensland Australia; 6 Bond University, Gold Coast, Australia; 7 Faculty of Health Sciences and Medicine, Bond University, Gold Coast, Australia

**Keywords:** Translational simulation, Input-process-output model, Operational framework, Quality improvement, Human factors/ergonomics, Healthcare simulation, In situ simulation

## Abstract

This article describes an operational framework for implementing translational simulation in everyday practice. The framework, based on an input-process-output model, is developed from a critical review of the existing translational simulation literature and the collective experience of the authors’ affiliated translational simulation services. The article describes how translational simulation may be used to explore work environments and/or people in them, improve quality through targeted interventions focused on clinical performance/patient outcomes, and be used to design and test planned infrastructure or interventions. Representative case vignettes are used to show how the framework can be applied to real world healthcare problems, including clinical space testing, process development, and culture. Finally, future directions for translational simulation are discussed. As such, the article provides a road map for practitioners who seek to address health service outcomes using translational simulation.

**Supplementary Information:**

The online version contains supplementary material available at 10.1186/s41077-021-00160-6.

## Introduction

Translational simulation is a functional descriptor of healthcare simulation whose purpose is to directly improve patient care and healthcare systems, through *diagnosing* safety and performance issues and delivering simulation-based *interventions*. The focus is on the *purpose* of the simulation activities, irrespective of the location, modality, or content [1]. Many examples of translational simulation activities and applications have been described, mostly as context-specific case studies (see below). However, guidance for how practitioners and organisations can enact translational simulation in everyday practice is lacking.

In this article, we describe a ‘road map’ for practitioners using translational simulation to address health service and patient-oriented outcomes. Our advice is based on a critical review of existing literature and reported examples, combined with the experiences of four translational simulation services in two countries (Australia and Canada), who have collectively delivered more than one thousand translational simulation activities over an 8-year period. We apply our suggested operational framework to three hypothetical examples and provide a versatile toolkit for practitioners of translational simulation.

## Translational simulation in action

### Translational simulation may be used to explore work environments and/or people in them

Diverse techniques can be employed to review ‘performance shaping factors’ in healthcare—at the level of individual, team, technology, work environment, and system [2, 3]. Examples include task trainers to study procedural skill performance, scenario-based immersive simulations to study team performance, simulated patient role plays to review communication, or computer modelling simulation to examine patient flow through an emergency department. Simulations conducted within the actual care setting—in situ simulation (ISS)—can be used to evaluate system performance and identify latent conditions that pose patient safety threats [4].The issues identified through this approach frequently relate to equipment, medication, physical space usage, and call systems [5]. In parallel, exploration may also occur within team relationships, roles, and culture [6]—equally important contributors to performance and safety in healthcare.

### Translational simulation may improve quality through targeted interventions focused on clinical performance/patient outcomes

The clearest examples are simulation interventions designed to improve measurable performance targets—e.g. time to thrombolysis in stroke care [7], time to computed tomography (CT) scan for trauma patients [8], resuscitation outcomes [9], teamwork in trauma [10], or success during intubation [11]. The methods may include dedicated educational programmes for individual and team performance—part task training for procedural skills, immersive simulations for team-based tasks, combined with practising in situ—as patient outcomes are dependent on individuals and teams performing within complex systems and departmental interfaces. Simulation design requires a clear objective and appreciation of the relative benefits of various simulation modalities that may support patient-oriented improvements, while remaining feasible and cost effective [1, 12].

Translational simulation activities may be diagnostic (determining what problems exist and their characteristics), interventional (providing solutions to problems), or a combination of the two. For example, the positive unintended change from ‘diagnostic’ trauma simulations may heighten awareness related to issues within the organisation’s staff and promote a collaborative culture [13]. Finally, improvements may also occur by highlighting successes during translational simulation activities, for instance by identifying effective workplace practices used by skilled clinicians and embedding them in standardised processes [14].

### Translational simulation may be used to design and test planned infrastructure or interventions

The re-creation of healthcare environments can provide opportunities to test the feasibility, safety, acceptability, or effectiveness of planned changes [2].Testing new healthcare facilities through simulation can trial workflows, address ergonomic issues, and identify latent safety threats before ‘go live’ [15–19]. This testing may include tabletop mock-ups and full scale recreations of facilities, and involve individuals or teams ‘working’ within these test environments. The approach requires more than a single ‘event’, but rather an integrated programme for testing and data collection. Petrosoniak et al. propose a ‘design thinking’ approach—a suite of simulation techniques that emphasises end user engagement—to iteratively test and improve upon changes [19]. Similar iterative approaches to testing and embedding identified system issues were used during the coronavirus disease 2019 (COVID-19) pandemic, when many healthcare workflows and practices had to be rapidly adjusted to minimise infection risks [20–22]. Simulation strategies helped to explore risks of COVID-19 transmission in current practices and to test the feasibility and effectiveness of planned changes designed to reduce risks at the individual, team, and system level. ‘Work as imagined’ strategies [23] that had intuitive appeal—e.g. Perspex boxes to protect airway teams from exposure to COVID-19 during intubation [24, 25]—were not always feasible or effective when tested in simulated practice.

## Case vignettes: problems to solve

Three case vignettes of translational simulation projects are presented below, based on real experience. Their purpose is to prompt the reader to consider how they might seek to address the problems posed, before reading on. We then describe an operational framework for translational simulation, during which the reader may wish to reflect on the case vignettes. Finally, we revisit the case vignettes to show how relevant aspects of the framework are applied in those contexts.

### Case 1: clinical space testing—new trauma bays

An academic hospital and trauma centre requires a new clinical environment for the emergency care of trauma patients. The institution planned for three trauma bays based on projected estimates of trauma volumes. The architect’s initial design borrowed many elements from the existing space. Clinicians with translational simulation experience who work in the trauma centre suggest further development using a design thinking approach coupled with simulation-informed clinical design.

### Case 2: process development—an airway emergency protocol for electroconvulsive therapy (ECT)?

A hospital is planning an ECT service, involving teams from anaesthesia and mental health, working in a newly built facility. The anaesthesia team are concerned about the possibility of airway emergencies in this ‘remote’ (i.e. non-operating theatre) context and suggest conducting simulations to test the environment, equipment, and the proposed ‘ECT airway emergency protocol’. The project is referred to the hospital’s translational simulation service who agrees that a translational simulation approach will help define the problem and is likely to be beneficial in identifying and addressing issues affecting ECT service provision in the new facility.

### Case 3: culture—postpartum haemorrhage (PPH)

A hospital is trying to improve care of women who suffer major PPH. A recent Coroner’s case has prompted action, and workplace surveys suggest that culture is a problem in the maternity unit, as are relationships between birth suite, blood bank, and operating theatre staff. Staff have suggested a new guideline is needed, including a handover proforma and an ‘obstetric haemorrhage respond’ call process. The institution engages an external consultation service to develop a translational simulation strategy to improve patient care.

## Translational simulation framework

### Guiding principles

**Fig. 1:**
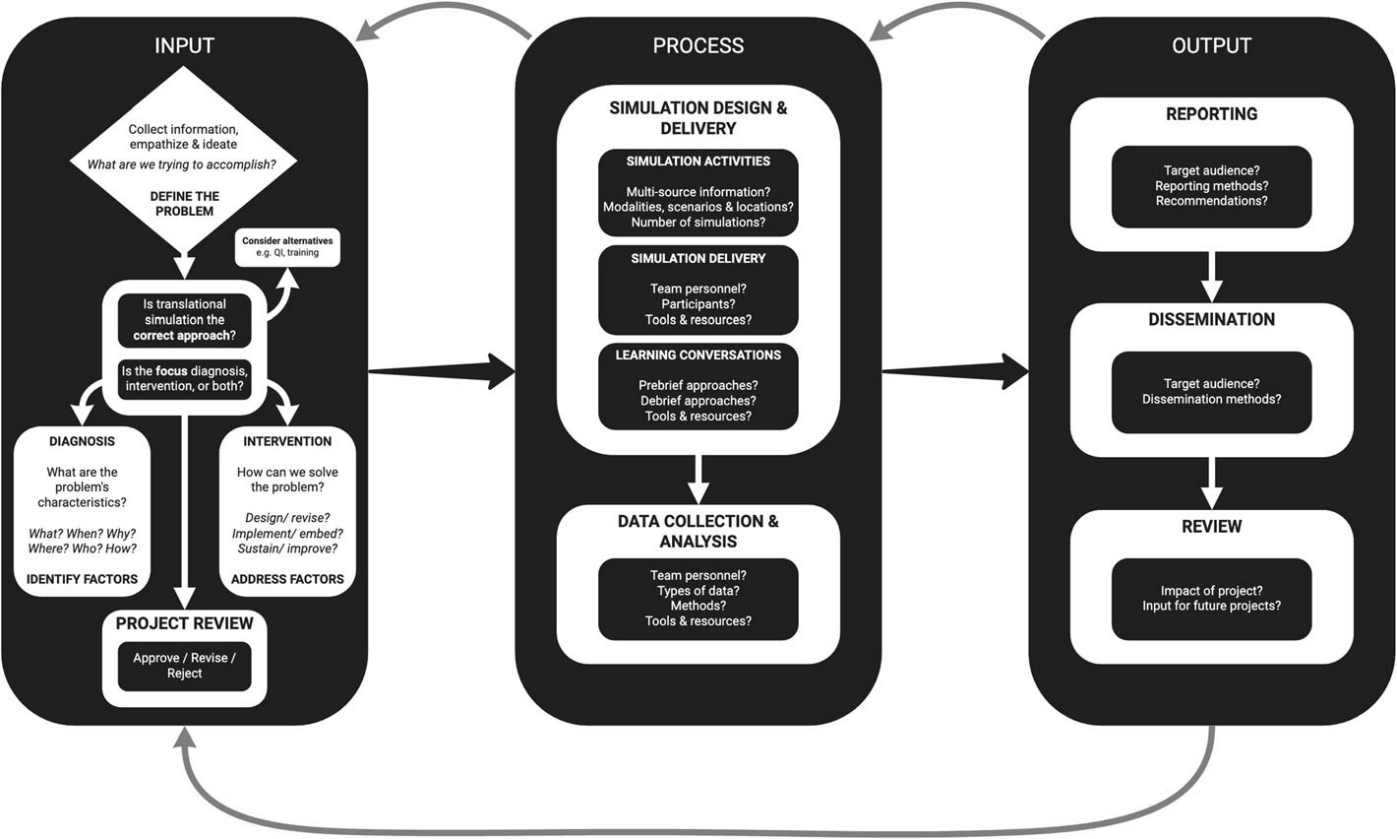
Input-process-output framework for translational simulation

We propose that translational simulation can be conceptualised operationally in terms of an input-process-output (IPO) framework (Fig. 1). IPO models are widely used in fields such as the study of team effectiveness and quality management [26, 27]. The guiding principles for our framework are that translational simulation requires the following:

1. A systems approach. Unlike traditional simulation-based education, which is focused on learning by individuals and small groups, translational simulation can promote *organisational* learning [28] by targeting improvements in systems’ components and their relationships [29]. Translational simulation strategies are likely to have greater impact when they are integrated with an organisation’s clinical governance and quality improvement processes, redesign and capital work planning, education and training, equipment procurement, and public relations. How this is achieved varies according to the context. In established systems, referrals may be made to a formalised translational simulation consultation service [30]. In the absence of an established programme, key representatives from each organisational unit may need to be approached on an ad hoc basis, and staff with relevant simulation expertise may be assigned by the organisation’s executive to key projects, such as the transition to electronic health records or capital works development.
2. Stakeholder involvement and participatory design. Effective translational simulation is inherently collaborative and benefits from involvement of the right stakeholders at the right time, including healthcare consumers [31]. Success often results from participatory design and co-creation, especially when the goal is to design new clinical spaces and new processes of care that impact multiple interprofessional teams and services. Examples of important stakeholders to consider including are listed in Table 1.
3. A strategy, not an event. Healthcare improvement requires an iterative approach. The IPO framework may create the illusion that translational simulation is rigid, linear, and stepwise in nature. In reality, new information is continually obtained as the translational simulation strategy develops, leading to iterative refinement of the input, process, and output phases.
4. Disciplined focus. While translational simulation activities may have elements of both diagnosis (determining what problems exist and their characteristics) and intervention (providing solutions to problems), goals are more likely to be achieved if they are narrow, specific, and well communicated to those designing and participating in the translational simulation activities.
5. Functional task alignment [13]. Specific simulation techniques and design choices (mannikins, simulated patient methodology, location, format, equipment) should be chosen according to how they align with the objectives of the translational simulation strategy.

**Table 1:** Considerations for the “Input phase” of translational simulation

Examples of stakeholders to involve	
Process experts	Simulationists (design, delivery, and debrief), simulation technologists, designers, change agents (ideally with translational simulation experience)
Content experts	Interprofessional clinical experts, non-clinical experts (e.g. human factors/ergonomics, design and planning, engineers, information technology, hospital services)
Frontline workers	Healthcare professionals who interface with the clinical problem as part of their daily work and understand ‘work as actually done’
Leadership	Department heads, hospital executive, institutional committees, clinical governance
Consumers	Patient advocates, people who can share patient experience
**Sources of information useful for defining the problem** • Prior translational simulation outputs • Benchmarking and standards • Organisational priorities • Clinical governance issues (e.g. sentinel events, incident monitoring systems) • Key performance indicators (KPIs) and performance metrics • Direct observation of relevant personnel and processes • Interviews, focus groups and surveys of relevant personnel • Artefact analysis (e.g. guidelines, cognitive aids, meeting minutes) • Capital works plans and designs • Engineering and/or ergonomic assessments • Brainstorm sessions and workshops involving relevant personnel • Government or institutional policy documents • Public health advice on emerging threats (e.g. pandemics)	
**Factors that influence the value of a translational simulation project** • Institutional priorities • Patient-oriented outcomes • Return on investment (ROI) (impact, efficiency, financial) • Impact on patient and staff experience • Local team support for project • Follow-up plan to show impact • Available resources (simulation and clinical) • High-value reporting strategy • Institutional readiness for change • Opportunity for research (requiring ethics approval) • Opportunity cost (what other translational simulation projects could be done?) • Safety (risk mitigation of unintended consequences)	

### Input phase

The ‘input phase’ of translational simulation has four main components: (1) define the problem; (2) consider if translational simulation is an appropriate approach to the problem; (3) determine if the focus of translation simulation is diagnostic, intervention, or both; and (4) project review. Ideas for projects may originate from referrals to a consultation service [30], or may be self-generated by teams capable of initiating translational simulation activities within their own clinical and non-clinical units.

1. *Define the problem.* In the tradition of Berwick’s Plan-Do-Study-Act (PDSA) cycle for quality improvement [32], we need a clear answer to the question ‘what are we trying to accomplish?’, although this may be iteratively revised over time. Clarity about whether our focus is diagnostic, interventional or both will support better design and delivery of translational simulation. Multiple sources of information are used to help define the problem (see Table 1). Numerous healthcare performance domains and metrics can be targeted by translational simulation [1], and though quantitative measures are enticing, we are reminded by William Bruce Cameron that ‘not everything that can be counted counts, and not everything that counts can be counted’ [33].

2. *Ask* ‘Is translational simulation an appropriate approach?’ Translational simulation may not be the most efficient, feasible, or cost-effective solution, nor is it likely to be a singular approach to complex healthcare challenges. Exploration and optimisation of organisational performance may be achieved by applying quality improvement methods to actual work environments and practices. Specific education and training is more appropriate when the focus is individual learning. Translational simulation is most effective when using the ‘real world’ as a test bed is not feasible or ethical [34], and when resource use can be justified by likely benefit to patients and systems.

3. *Project review.* Even when, in principle, translational simulation is an appropriate strategy for tackling a defined problem, a more granular project review should consider four important questions. Based on the answers to these questions, proposed translational simulation projects can be accepted (and prioritised), refined, or declined.

i. Continue the PDSA approach and ask, ‘is translational simulation likely to lead to an improvement?’. This requires clarity about measures of success—how to determine whether a change has led to an improvement and which elements of the change were effective. This will guide approaches to data collection and analysis.
ii. ‘Is the translational simulation project worth the cost?’ Factors that influence the value of a translational simulation project are shown in Table 1.
iii. ‘Will the translational simulation activity have unintended consequences?’ Safety and integrity of real clinical environments and systems may be threatened by techniques such as in situ simulation delivery [35]. More fundamentally, there may be unintended outcomes or ‘balancing measures’ to be considered [36]. Solutions to one problem can create new ones.
iv. “Is there capability to deliver the translational simulation project with the personnel, resources, and time available?” Some projects with limited scope can be developed and delivered over a few days, but ambitious projects may require months of planning.

### Process phase

The ‘process phase’ of translational simulation has three main components: (1) simulation design and delivery, (2) data collection, and (3) data analysis. Examples of tools and techniques for data collection and analysis are provided in Table 2. Any tools and techniques should be adapted as required for the chosen purpose and context.

1. Simulation design and delivery. Well-established principles of scenario design and delivery for educationally focused simulation [48] can be applied and adapted to translational simulation.

i. Scenarios to support translational simulation activities are best developed using multi-source information involving appropriate stakeholders (see Table 1). One or multiple simulation modalities and scenarios may be required to address the chosen aspects of the problem. The modality and location of the simulation activities will vary depending on their purpose, guided by the principle of functional task alignment in a similar way to educationally focused simulation [49]. Scenarios should be peer reviewed and piloted prior to use.
ii. Simulation delivery requires appropriate personnel—facilitators, debriefers, content experts, simulation operations and technical specialists, as well as support staff to help prepare and reset scenarios and equipment. Approaches will vary in different contexts. A common approach involves a dedicated simulation team engaging with clinicians from the relevant areas and with internal or external consultants with identified expertise in the issue. Ideally, the simulation participants are authentic clinical teams familiar with the problem being addressed and the context of the work environment. Any observers are carefully chosen to maintain the psychological safety of the participants and promote useful data collection (see below). Commonly, matched observers (e.g. a surgical nurse observing surgical nurse roles) and additional content experts (e.g. an information technology expert to observe electronic health record use) are valuable. The use of templates and checklists are recommended to ensure safety and efficiency of simulation design and delivery.
iii. Learning conversations. As with any simulation activity, prebriefing and debriefing are critical for participants and observers to engage with the translational simulation process and to optimise the data collected. The prebriefing should clearly outline the purpose and objectives of the activity, what will be done with the findings, the roles of participants and observers, and establish a ‘safe container’ for everyone involved [50]. It should reinforce messages in any pre-reading sent to participants prior to the event. The debrief process should be tailored to purpose and the constraints of real clinical teams and time pressures. Rich data is often derived from well-conducted debriefs of participants and observers, so participants should understand their role in the wider translational simulation strategy.

2. Data collection. Data collection should focus on measures specific to the translational simulation targets and may be qualitative, quantitative, or both. Effective data collection is facilitated by carefully selected observers, the use of observer tools, and a multi-modal approach to monitoring performance. Observer tools (see Table 2 for examples) may be based on conceptual frameworks, evidence-based principles, validated assessment tools, and/or process-orientated events. Direct observation can be supplemented by remote observation and recording using video communication platforms, advanced monitoring modalities such as motion tracking [5] and eye movement tracking [40], and post-event data collection.

3. Data analysis. The data analysis team should involve complementary process experts (both in translational simulation and the chosen analysis approach) and content experts (to help make sense of the data in clinical context). The analysis approaches used are aligned with the type of data collected and the overall translational simulation strategy.

**Table 2:** Considerations for the “Process phase” of translational simulation. Examples of tools and techniques for data collection and analysis*

Direct observation	*Observers* selected for expertise; may need training in assessment tools *Assessment tools* Procedure-specific assessment tools, e.g. arterial blood sampling [37] Global rating scale for procedural skills [37] Teamwork, e.g. Team Emergency Assessment Measure (TEAM) [38] Time-to-event, e.g. time to CT scan for trauma patients [8] Safe design goals observer tool [39] *Ethnographic observation* [6]
Monitoring	Video and/or audio recording and streaming Motion tracking [5] Eye movement tracking [40] Other ergonomic assessment tools (e.g. heart rate monitoring, strain measurements) [41]
Learning conversations	*Debrief approaches* Rapid cycle deliberate practice [42] (can be modified to improve processes as well as individual performance) Systems-focused Promoting Excellence and Reflective Learning in Simulation (PEARLS) framework [43] SAFEE debriefing tool [44] (based on evidence-based design principles) Pluralist walkthrough [41] with iterative discussions ‘Brainstorm’ sessions [45, 46] (e.g. with participants having the opportunity for quiet reflection and labelling the environment and equipment with sticky notes as a starting point for discussion) *Documentation* Whiteboards, sticky notes, and photography Technology-enhanced (e.g. Trello^TM^ [47] as a virtual ‘sticky note board’) Video recording, audio recording and transcription
Post-event data	Review of video recording, audio recording and transcripts Interviews and focus groups [45, 46] Surveys, e.g., Relational Coordination Survey [6] Artefact analysis (e.g. guidelines, cognitive aids, checklists, debrief reports) [41]
Analysis	*General* [42, 44, 45] Qualitative analysis of interviews, focus groups, surveys and artefacts Statistical analysis of quantitative data (e.g. time to completion, survey data) *Human factors/ergonomics* Failure Modes Effect Analysis (FMEA) [15, 39] to risk stratify threats Hierarchical task analysis [41] to understand task steps Cognitive task analysis [41] to understand cognitive processes during tasks Charting techniques [42, 44, 45], e.g. process charts, decision action guidelines Mental workload assessment techniques [41], e.g. NASA Task Load Index Situation awareness measurement techniques [41] Team assessment methods (see also above) Interface analysis [41], e.g. walkthrough analysis Performance time assessment techniques [41], e.g. Critical Path Analysis Design techniques [41], e.g. rapid prototyping, think aloud protocols *Quality improvement* Gathering information [45, 46], e.g. stakeholder analysis, benchmarking Problem solving [45, 46], e.g. Five Whys Understanding variation [42, 44, 45], e.g. statistical process control Simulation-based Quality Improvement Tool (SQOIT) [15] Incident reporting and root cause analysis [45, 46] (e.g. latent threats identified by ISS) Cost-benefit analysis (60, 62)

*Templates and instructions provided in the cited references, with additional selected examples used by the authors provided in the online supplemental appendix

### Output phase

The ‘output phase’ of translational simulation has three main components: (1) reporting, (2) dissemination, and (3) review. Suggested reporting and dissemination approaches are shown in Table 3.

1. Reporting. How the findings of the translational simulation project will be presented, in what format, and to whom are considered. Findings are reported collectively, without reference to specific individuals so that participant confidentiality is maintained. Ideally, outcomes reported from translational simulation are integrated with performance monitoring processes across the organisation—requiring alignment of tools, measures, reporting cycles, and governance structures. The personnel who are accountable for any recommended actions must be clearly identified and assigned.

2. Dissemination. There may be a wide target audience for sharing outcomes and lessons from translational simulation activities—clinical staff and managers at the department level, leaders within the wider organisation, external bodies, the general public, or the research community. They each require different approaches to dissemination, both in terms of modality and information content.

3. Review. The impact of the project compared to planned goals is considered—this includes changes in environment/clinical space design, equipment, informatics, education and training, cognitive aids and checklists, policies and guidelines, and/or workflows (e.g. simplification, standardisation, automation and computerisation, and forcing functions). Does the project generate useful data or new questions for further translational simulation work, and what can be learned about how to improve techniques for future translational simulation?

**Table 3:** Considerations for the “Output phase” of translational simulation. This grid provides suggested reporting and dissemination methods (indicated by a “black star” symbol) according to the target audience of translational simulation outputs. The information content should also be tailored to the target audience

	Local (department)	Wider organization	External bodies	Research community	General public
Meetings & education sessions	★	★			
Infographics & posters	★	★		★	
Social media, blogs, video & podcasts	★	★		★	
Email	★	★			
Clinical governance (guidelines, policies)	★	★	★		
Conference presentations			★	★	
Journal publication				★	
Public relations & news media		★	★		★

## Case vignettes: translational simulation in action

Having outlined an operational framework for translational simulation, we revisit our case vignettes to show the potential outcomes of applying this framework.

### Case 1: clinical space testing—new trauma bays

Concerns are voiced by clinicians about the functionality and usability of the initial design. The clinical end users and the design team are unable to reach consensus on the design functionality but agree that simulation testing may help them resolve their differences. Projected translational simulation costs are incorporated into the project budget as part of the necessary design and commissioning costs. An ad hoc translational simulation team is formed, led by two trauma clinicians with experience in using in situ simulation to identify latent safety threats, combined with five staff from the hospital’s simulation centre and a member of the design team. Simulations begin in the existing trauma bay to better understand the teams, their workflows, and their needs. Regular updates are presented to the project team during the translational simulation activities. Direct observation and movement tracking within the space [5] finds that the new space cannot accommodate an expansion to three trauma bays and that two bays are ergonomically optimal. Tabletop simulations followed by simulations within mock-ups of the space confirm other previously identified latent safety threats [51]. The process follows the Agency for Healthcare Research and Quality (AHRQ) evidence-based safe design principles [40], and data from observations and debriefings are documented on the Simulation-based Quality Improvement Observation Tool (SQIOT) [14] by both observers and simulation facilitators. Data from the SQIOTs are collated, risk rated, and reported on a Healthcare Failure Modes Effects Analysis (HFMEA) summary report [15, 39]. Recommendations are provided in the HFMEA summary report, which is submitted to the project team and organisational leadership for action. Using this process, several ‘blind spots’ within the new space are identified that translate to tangible improvements. Specific changes include the addition of multiple vital sign monitors surrounding the clinical space, modular procedure carts to mitigate space limitations and floor markings to better delineate the clinical care environment. The presence of architects during the process enables a more efficient design process that meets the clinician and patient needs. Importantly, final state simulations validate the functionality of the implemented changes and ensure that the new clinical space is not first tested on patients.

### Case 2: process development—an airway emergency protocol for electroconvulsive therapy (ECT)

The translational simulation team of six people is led by a physician with expertise in translational simulation and includes simulation educators and technologists, and local champions from the anaesthesia and mental health units. Focus groups, involving staff from anaesthesia and mental health, are conducted to understand the concerns these stakeholders have about the opening of this new service. The anaesthesia team identifies concerns about the possibility of airway emergencies in this ‘remote’ (i.e. non-operating theatre) context. A translational simulation strategy is designed and delivered in the new physical space prior to opening, with simulated patient actors and the relevant healthcare provider teams. Key design elements include a structured data collection process during the simulation and debrief—developed through a series of meetings with anaesthetists, mental health staff, and the hospital risk management unit. The decision is made to test the basic workflow with simulated patient actors prior to scenarios involving critical airway incidents. Minor issues are identified with the physical environment, but feedback from the simulated patients, who are involved in the debriefs, is of major concern. The proximity of the ECT suite next to the waiting patients means that they can hear all of the activity of ECT being delivered, and they find this frightening. The waiting area is moved prior to the service commencing. Subsequent scenarios testing the specific airway emergency protocol result in minor, iterative changes. Short (<10 min) ‘mental rehearsal’ simulations for airway crises are designed and run weekly at the start of the ECT list. An overview of the ECT simulation programme is presented at the hospital grand rounds. Interest from external groups results in the development of a multi-disciplinary course for ECT providers that has modest commercial success.

### Case 3: culture—postpartum haemorrhage

The external translational simulation consultants work with an interprofessional obstetrics team from the host institution to define the problem with patient care. The institution undertakes an audit of PPH cases, together with the coronial findings and the root cause analysis of the critical incident, and baseline measure of blood transfusion rates for major PPH. A Relational Coordination Survey [6] is conducted to examine the strengths and weaknesses between and within teams involved in major PPH cases, which informs focus group discussions about how to improve performance. As a result, a translational simulation strategy is formulated to design, test, and embed a series of interventions, including a simplified guideline for major PPH, a ‘STOP’ handover moment after arrival in the operating theatre, and an ‘Obstetric Haemorrhage Respond’ call process. Desktop and walkthrough simulations are used to refine a prototype guideline. In collaboration with the hospital quality improvement unit, more than 40 live scenario-based simulations are used to further test and embed the guidelines, supported by educational sessions, infographics, and presentations at ward in-services. During these simulations, further ‘diagnoses’ are made—the need for a hospital massive transfusion protocol and the need for a clinical event debriefing programme within maternity services—leading to additional translational simulation projects. After 6 months, relational coordination is remeasured, and workplace surveys show an improvement in staff culture. Transfusion rates in PPH show a small, non-statistically significant decrease.

## Future directions for translational simulation

### Demonstrating return on investment

Translational simulation programmes must provide value to healthcare organisations, given the intensive resources required. Demonstrating return on investment (ROI) is a priority for any improvement or educational activity, and frameworks exist for determining ROI in simulation [52, 53]. Sometimes the ROI is obvious, such as when a simulation activity identifies that expensive equipment is not fit for purpose and should not be purchased. Growth of translational simulation is partially predicated on demonstrable ROIs. During the design of any translational simulation programme, data collection, data analysis and outcomes can be aided by a collaboration with health economists.

### Connecting translational simulation, quality improvement, and human factors/ergonomics

Integrating translational simulation strategies with existing quality improvement and human factors/ergonomics (HFE) approaches and communities of practice is necessary to maximise benefits and reduce redundancy and conflict. This requires deeper understanding of the techniques used by the different communities and more aligned tools, governance structures, professional development opportunities, and research agendas [54]. HFE approaches are especially useful because they are design-driven, take a systems approach, and focus on optimising both system performance and human wellbeing [55]. Unfortunately, capacity and expertise for HFE approaches have been underdeveloped in healthcare [56, 57] despite evidence of their effectiveness [58, 59].

### Building capacity for translational simulation within health services

As a nascent field, translational simulation has been embraced by enthusiasts, but few mature exemplars exist. The natural evolution in many organisations is for enthusiastic practitioners to carry out translational simulation activities on their own time using existing resources. This involves co-opting simulation-based education resources, then using the outputs of these activities to convince their organisation of the value of the approach (e.g. ROI) and obtaining more resources to make translational simulation sustainable. Implementing translational simulation requires trained staff, governance structures, adequate technology and physical resources, and a profile within the organisation and/or broader health system. There may be a role for external consultation services in assisting organisations that lack translational simulation expertise or resources. Guidance on simulation safety [35], in situ simulation techniques [60], faculty development, tools for simulation-based clinical systems testing [39], and debriefing systems-focused simulation [43] has been published. However, more robust evidence is required to support the development of the field and its demonstrable value for patient safety and outcomes.

### Translational simulation and the COVID-19 pandemic

The COVID-19 pandemic presented health services with the need for rapid and high stakes change to processes, workflows, teamwork, and physical spaces to prevent the spread of infection. Well-established translational simulation programmes strongly aligned with health service priorities were able to nimbly develop strategies to support these changes [20–22, 61], including an outstanding example delivered at a provincewide level in Canada [62]. Given the likelihood of ongoing need for healthcare change and redesign in response to COVID-19, translational simulation approaches will remain critical. However, the pandemic has also added significant constraints on simulation delivery, and translational programmes will need to develop solutions for ‘COVID-safe’ design, delivery [63], and debriefing [64].

## Conclusion

Translational simulation is an emerging strategy for improving health service performance and patient outcomes. Our iterative input-process-output model offers an operational approach to applying diverse aims, approaches, and tools to solve real world problems. Our guiding principles and practical techniques draw on practice in simulation-based education, human factors/ergonomics, and quality improvement, but recognise their application occurs across diverse contexts. Translational simulation remains a nascent but promising approach to supporting solutions for the growing complexity of healthcare.

## Supplementary Information

**Additional file 1:.** Supplemental appendix

## Data Availability

Not applicable

## Abbreviations

AHRQ: Agency for Healthcare Research and Quality
COVID-19: Coronavirus disease 2019
HFE: Human factors/ergonomics
IPO: Input-process-output
ISS: In situ simulation
KPIs: Key performance indicators
QI: Quality improvement
ECT: Electroconvulsive therapy
HFMEA: Healthcare Failure Modes Effects Analysis
PEARLS: Promoting Excellence and Reflective Learning in Simulation
PPH: Postpartum haemorrhage
PDSA: Plan-Do-Study-Act
ROI: Return on investment
SQIOT: Simulation-based Quality Improvement Observation Tool
TEAM: Team Emergency Assessment Measure
