# Supplementary material for: Translational simulation: from description to action
Source: Adv Simul (Lond). 2021 Mar 4;6:6. doi: 10.1186/s41077-021-00160-6 (PMC7930894; doi:10.1186/s41077-021-00160-6)
Supplement: Supplementary file 1 — Additional file 1:. Supplemental appendix [file 41077_2021_160_MOESM1_ESM.docx]

Supplemental appendix

Examples of templates and tools used by some of the author’s simulation services. Numerous other data collection and analysis tools and instruments can be found in the references cited in the examples listed in Table 2 of the main article.

- [Simulation event report template](https://drive.google.com/file/d/1-oV8xdYsPboViLBeBOllwPkRj5n3lUKi/view?usp=sharing) (Gold Coast Health Simulation Service)
- [Simulation scenario template](https://drive.google.com/file/d/1SdaWY-06wypeN5_C2xcL9ft-9RVZ5VdN/view?usp=sharing) (Gold Coast Health Simulation Service)
- [End of Year activity report example](https://drive.google.com/file/d/1AzWqRw44SSnNz01RFOVTc5ayQXfGC9Gc/view?usp=sharing) (Gold Coast Health Simulation Service)
- ['After Hours' STEMI to Cath Lab translational simulation scenario](https://docs.google.com/document/d/1NXx34bygGiKU5seGXdjSyAu2H4YQhL0nwWYd6v1ZTgk/edit?usp=sharing), including pre-simulation checklist (Alfred Health Centre for Health Innovation)
- [Clinical Space Testing Modified HFMEA Report Template](https://docs.google.com/document/d/1gX37L7sdve6eTC0BIO-MSFObj3f4Y3EltVIisA0TqFg/edit?usp=sharing) (Alfred Health Centre for Health Innovation)
- [In situ simulation safety briefing checklist](https://healthcaresimulationsafety.files.wordpress.com/2019/01/in-situ-simulation-safety-checklist-brazil-purdy-december-2018.pdf) (Victoria Brazil and Eve Purdy)
